# Comparison of Venous Thromboembolism after Total Artificial Joint Replacement between Musculoskeletal Tumors and Osteoarthritis of the Knee by a Single Surgeon

**DOI:** 10.1371/journal.pone.0158215

**Published:** 2016-06-28

**Authors:** Dong Fu, Yiqiong Zhao, Jiakang Shen, Zhengdong Cai, Yingqi Hua

**Affiliations:** 1 Department of Orthopedics, Shanghai First People's Hospital, School of Medicine, Shanghai Jiaotong University, Shanghai 200080, China; 2 The tenth People's Hospital affiliated with Tong Ji University, Shanghai 200072, China; IIBB-CSIC-IDIBAPS, SPAIN

## Abstract

The purpose of this study was to compare and evaluate the event of VTE (Venous Thromboembolism Event) after total artificial joint replacement between two groups diagnosed with either musculoskeletal tumors or osteoarthritis (OA) of the knee. From 2004 to 2014, a total of 1,402 patients (308 in tumor group, 1,094 in OA group) were involved in this study. The rate of asymptomatic DVT (Deep vein thrombosis) was significantly higher in tumor group when compared with OA group. Though both the incidence of symptomatic DVT and PE (Pulmonary embolism) were slightly higher in tumor group, no significant difference was detected. Tumor patients suffered an almost equal risk of VTE compared with OA patients except a higher rate of asymptomatic DVT after total artificial joint replacement. For patients with tumor, no significant association was observed between any potential risk factor and DVT.

## Introduction

Postoperative venous thromboembolism, which encompasses deep vein thrombosis and pulmonary embolism, has been recognized as a potentially life-threatening complication[1]. In England, more than 25,000 people die of venous thromboembolism each year for various kinds of surgeries[2]. Orthopedic surgery especially that involving lower limbs has always been regarded as a risk factor for the development of venous thromboembolism[3]. Total knee arthroplasty and total hip replacement which are widely used for patients with degenerative osteoarthritis have been proved as the most common causes for DVT in orthopedic surgeries. On the other hand, with the development of surgical technology, prosthesis design and chemotherapy, limb salvage and retention of function are possible for patients with a musculoskeletal tumor [3–5]. As the first treatment choice currently, more and more limb salvage surgeries have replaced amputation for patients with tumors involving the knee joint. The relationship between prosthesis reconstruction of limbs and thromboembolism has also drawn much more attention. Previous oncological studies demonstrated that patients diagnosed with tumors of different locations would have at least twice the risk of developing a post-operative DVT and three times the risk of developing a fatal PE than patients without cancer[6–8]. However, there was little information in the literature specifically indicating that patients who had received the knee reconstruction surgery for treatment of bone and soft tissue tumors would have a higher risk of VTE [9–14]. We performed one retrospective study of a cohort of patients diagnosed with either musculoskeletal tumors or osteoarthritis of the knee in order to evaluate the risk of symptomatic/asymptomatic deep venous thrombosis and symptomatic pulmonary embolism after artificial joint replacement between two groups. We hypothesized that patients with musculoskeletal tumors might be at a particularly high risk for venous thromboembolism.

## Method

### Participants

We retrospectively reviewed the clinical records from January 2004 to January 2014 and selected patients who were scheduled for artificial knee joint replacement surgery with confirmed diagnosis of osteoarthritis or musculoskeletal tumors. Patients were excluded if they had (1) a superficial mass which didn’t involve the underlying muscle or bone, (2) a bilateral knee replacement, (3) non-osteoarthritis (rheumatoid arthritis, hemophilic arthritis, suppurative arthritis et al), (4) a detection of DVT before surgery, (5) been lost to follow-up less than three months after treatment. As a result, a total of 1,094 patients (573 females and 521 males) who had undergone total knee arthroplasty for osteoarthritis and 308 patients (168 females and 140 males) who had undergone prosthesis reconstruction of the knee for musculoskeletal tumors by one skilled orthopedic surgeon were enrolled. It was important to avoid damage to endometrium by careful manipulation intraoperatively. The tourniquet was applied routinely from incision to wound closure. Adequate rehydration was required intraoperatively and postoperatively to avoid blood concentration. The wound was bandaged with suitable pressure and early active regular exercise was advised for all patients. None of the patients had received mechanical preventive methods like intermittent lower inflation pressure pump. From 2004 to 2009, all patients received low molecular weight heparin (0.4ml /4100AxaIU/qd) as the anticoagulant therapy. After then, Rivaroxaban (10mg/qd) was applied gradually as its oral convenience. This study was approved by the ethics committee of Shanghai First People's Hospital, School of Medicine, Shanghai Jiaotong University. However, the written informed consent was not obtained and patient records/information was anonymized prior to analysis.

### Data collection

Data on patient demographics, pathological type, primary or metastatic tumors, anatomical location, complications, presence of additional risk factors for VTE were gathered.

### Clinical Management and Assessment of VTE

Standard Doppler ultrasonography of the lower limbs was conducted routinely for each patient to screen all non-symptomatic DVT before discharge and at the last follow-up. If a thrombosis was clinically suspected with the following clinical features (swelling, pain, positive Homen’s sign, erythema and fever), earlier ultrasound examination and venography were applied. If one patient had presented symptoms such as shortness of breath, tachycardia, hypoxia, hypotension or chest pain, we would apply the emergency inspection of computed tomography/angiography of the chest to screen a symptomatic PE. All the above mentioned procedures were completed by experienced doctors to ensure the precise incidence of VTE.

### Statistical Analysis

For the comparison of incidence of DVT and PE between two groups, *χ*^2^ test was used. *P*<0.05 was considered statistically significant. T test was used to check whether the data met normal distribution. VTE and Non-VTE patients were compared and analyzed with reference to age and gender, BMI (Body Mass Index), histological type of tumors (benign or malignant), absence of metastases, additional risk factors for thromboembolism using with multivariate cox regression analyses All the statistical analyses were performed using SPSS 17.0 (College Station, Texas 77845 USA).

## Results

During the ten years (from 2004 to 2014), a total of 1402 patients (308 in tumor group, 1094 in osteoarthritis group) were included in this study. Information of demography of two groups was showed at Table 1. The mean age was 38y and 67y respectively with a widely ranged follow-up from 3 to 118 months. A total of 67 and 170 VTE events were detected for tumor and OA group respectively. In tumor group, the number of Asymptomatic DVT, Symptomatic DVT, Symptomatic PE happened before discharge was 46, 13, 5 respectively. Another 3 Asymptomatic DVT events were detected during follow-up. Among the 308 tumor cases, 175 accepted chemotherapy and 84 accepted radiotherapy. The incidence of VTEs were 22.9% (40/175) and 25% (21/84) respectively. In Osteoarthritis group, the number was 113, 33, 13 for Asymptomatic DVT, Symptomatic DVT and Symptomatic PE before discharge. 11 Asymptomatic DVT events were detected during follow-up.

**Table 1:** Demographics of two groups.

	Musculoskeletal tumors	Osteoarthritis	*p*
Number of cases	308	1094	
Age (range)	38year (38.0±17.14)	67year (67.1±7.8)	<0.01
Female/Male	168/140	573/521	0.519
Body mass index (range)	18.8 kg/m2 (18.8±2.83)	27.8 kg/m2 (27.8±5.4)	<0.01
Follow-up (range)	49 month (49.2±16.4)	56 month (55.9±18.2)	0.628

All data obeyed the law of normal distribution. Our patients’ population was somewhat heterogeneous with a larger proportion of younger cases in tumor group. Distal femur was found to be the most common tumor location in 232 patients (75.3%). 72 lesions were located at the proximal tibia and 4 invaded the proximal tibia from the distal femur. 27 patients had suffered a pathological fracture even under an extremely slight force. The most commonly diagnosed malignant tumor was osteosarcoma and the benign one was giant cell tumor. Of the 308 patients with musculoskeletal tumors, 38 lesions were proved to be metastatic from other origins and the remaining 270 cases had a diagnosis of primary tumor. (Table 2)

**Table 2:** Potential risk factors of VTE for patients with musculoskeletal tumors around the knee with multivariate cox regression analyses.

	Patients (No)	VTE (n,%)	B	SE	Wald	Sig	Exp (B)
**Demographic data**							
Age<60y	255	53 (21.2%)	-0.371	0.177	4.414	***0*.*055***	***0*.*690***
Age>60y	53	14 (26.4%)					
Female	168	37 (22.0%)	-0.127	0.134	0.903	***0*.*342***	***0*.*880***
Male	140	30 (21.4%)					
BMI<25kg/m^2^	284	60 (21.1%)	0.269	0.263	1.264	***0*.*261***	***1*.*344***
BMI>25kg/m^2^	24	7 (29.1%)					
**Tumour history**							
Benign	128	23 (18.0%)	0.262	0.524	0.249	***0*.*618***	***1*.*299***
Malignant	180	44 (24.4%)					
Bone tumor	82	17 (20.7%)	0.132	0.151	0.767	***0*.*381***	***1*.*141***
Soft tissue tumor	226	50 (22.1%)					
Primary	270	55 (20.4%)	0.033	0.199	0.028	***0*.*867***	***1*.*034***
Metastasis	38	11 (29.0%)					
Pathological fracture	27	7 (26.0%)	0.089	0.260	0.117	***0*.*732***	***1*.*093***
Non-pathological fracture	281	60 (20.0%)					
**Location**							
Distal femur	232	54 (23.3%)	0.009	0.149	0.004	***0*.*952***	***1*.*009***
Proximal tibia	72	12 (16.7%)					
Total knee joint	4	1 (25.0%)					
**Chemotherapy**							
Yes	175	40 (22.9%)	-0.150	0.530	0.081	***0*.*776***	***0*.*860***
No	133	27 (20.3%)					
**Radiotherapy**							
Yes	84	21 (25.0%)	-0.110	0.179	0.375	***0*.*540***	***0*.*896***
No	224	46 (20.5%)					

**VTE includes** symptomatic/asymptomatic deep venous thrombosis, symptomatic pulmonary embolism; DVT includes symptomatic/asymptomatic deep venous thrombosis.

The rate of asymptomatic DVT was significantly higher in tumor patients (16.0%, 49/308) compared with that of OA patients (11.3%, 124/1094) (*p* = 0.039). Even though the incidence of symptomatic DVT in tumor group (4.2%, 13/308) was higher than that of OA group (3.0%, 33/1094), no significant difference was detected (*P* = 0.89). In terms of symptomatic PE, like symptomatic DVT, a trend of higher incidence was found to be associated with tumor patients. However, there remained no significant difference (*p* = 0.567). For patients with symptomatic DVT, no significant difference was detected regarding death, infection, dysfunction, ICU treatment or longer hospitalization (more than 1 month) between two groups (Table 3). With the data available, none of the following indexes like gender, age ≥60 years, BMI>25kg/m^2^, malignant tumor, previous pathological fracture, histological type of tumor, tumor location chemotherapy or radiotherapy was found to be significantly associated with the occurrence of VTE or DVT (Table 2). For OA patients, gender, age ≥60 years, BMI>25kg/m^2^ failed to make a significant relation with the occurrence of VTE (Table 4). Analysis of group as a potential risk factor of VTE with multivariate cox regression showed no significant difference between two groups for symptomatic DVT and PE (Table 5). Additionally, we also made a subgroup analysis between two groups with matched age and BMI. Still, no difference was detected in any index (Table 6).

**Table 3:** Comparison of major complications between two groups.

	Tumor group	OA group	P	Kaplan-Meier
**Death**	2.2% (1/46)	0.09% (1/113)	0.496	0.521
**Dysfunction**	8.7% (4/46)	6.2% (7/113)	0.731	0.773
**ICU treatment**	6.5% (3/46)	5.3% (6/113)	0.719	
**Longer hospitalization**	6.5% (3/46)	7.1% (8/113)	1.000	

**Table 4:** Potential risk factors of VTE for patients with osteoarthritis with multivariate cox regression analyses.

Demographic data	Patients (No)	VTE (n,%)	B	SE	Wald	Sig	Exp (B)
Age<60y	180	23 (12.8%)	-0.121	0.147	2.114	*0*.*125*	*0*.*730*
Age>60y	914	147 (16.1%)					
Female	573	90 (15.7%)	-0.221	0.236	0.706	*0*.*347*	*0*.*630*
Male	521	70 (13.4%)					
BMI<25kg/m^2^	443	60 (21.1%)	0.169	0.632	0.864	*0*.*461*	*0*.*944*
BMI>25kg/m^2^	651	7 (29.1%)					

**Table 5:** Analysis of group as a potential risk factor of VTE with multivariate cox regression analyses.

	Musculoskeletal tumors	Osteoarthritis	Sig	Exp (B)
Asymptomatic DVT	16.0% (49/308)	11.3% (124/1094)	*0*.*039*	*0*.*628*
Symptomatic DVT	4.2% (13/308)	3.0% (33/1094)	*0*.*890*	*1*.*231*
Symptomatic PE	1.6% (5/308)	1.2% (13/1094)	*0*.*567*	*0*.*873*
Total VTE events	21.8% (67/308)	15.5% (170/1094)	*0*.*016*	*0*.*347*

**Table 6:** Analysis of matched group as a potential risk factor of VTE with multivariate cox regression analyses.

	Musculoskeletal tumors	Osteoarthritis	Sig	Exp (B)
Asymptomatic DVT	15.9% (12/76)	10.3% (75/725)	*0*.*042*	*0*.*654*
Symptomatic DVT	3.9% (3/76)	2.8% (20/725)	*0*.*737*	*1*.*056*
Symptomatic PE	1.3% (1/76)	1.1% (8/725)	*0*.*643*	*1*.*008*
Total VTE events	21.1% (16/76)	14.2% (103/725)	*0*.*012*	*0*.*236*

## Discussion

Even effective pharmaceutical thromboprophylaxis combined with mechanical methods were used clinically, it has been reported that approximately 10% to 15% patients undergoing total artificial joint replacement experienced subclinical VTE and 0.5% to 2.0% had symptomatic VTE after surgery[15, 16]. It has been reported that the risk of developing a post-operative DVT and a fatal PTE would increase by twice and three times respectively in cancer patients compared with those without cancer [7, 17]. As well, Shen et al also found an increased risk of VTE in cancer patients in an autopsy series study [18]. However, tumor lesions reported in most studies were commonly located in the genitourinary tract, the gastrointestinal tract, and the breast, few had been described specifically in bone and soft tissue [9,11,14,19]. To our knowledge, the present study firstly demonstrated the incidence of VTE in two groups (musculoskeletal tumors vs Osteoarthritis) with a relative large cohort of patients.

In the present study, we reported a 16% incidence of asymptomatic DVT, a 4.2% incidence of symptomatic DVT and a 1.6% incidence of symptomatic PE in 308 tumor patients. For OA patients, the number was 11.3%, 3.0% and 1.2% respectively. The overall incidence of clinically detected DVT events was similar to that seen in other groups of patients undergoing primary artificial joint replacement for musculoskeletal tumors and osteoarthritis of the knee [9–11, 20]. Except a significant higher incidence of asymptomatic DVT was showed in the tumor group, no significant difference was found in symptomatic DVT and symptomatic PE. Though the exact reasons are not clear, several factors may play a role in the development of a relative higher asymptomatic DVT rate in tumor patients. First, musculoskeletal tumors in the knee may destroy the endothelial function by blocking venous return from distal extremity mechanically or invading the veins directly which is always associated with the process of thrombosis [21–23]. Second, time required for reconstruction of tumor prosthesis is significantly longer than that of TKA. Prolonged operating time increases the risk of bleeding and other surgical complications including VTE [24]. Third, in order to acquire a clear surgical margin, removal of large segments of bone and soft tissue is necessary, which will always lead to a longer postoperative recovery periods[10]. In addition, it is likely that tumors have a systemic procoagulant effect as what mentioned above that there is a trend of higher incidence of VTE in cancer patients with extra-musculoskeletal location.

Fortunately, despite the higher incidence of asymptomatic DVT as well as the potential factors which may contribute to the development of DVT in tumor patients, only few asymptomatic DVT will cause serious symptomatic DVT and PT. A fact which we can’t ignore is that patients in tumor group with a lower BMI (18.8 kg/m^2^ vs 27.8 kg/m^2^) were much younger than those with OA. Previous studies have noted that increased age and high BMI are two strong predictors for development of DVT [25]. The younger and with lower BMI the patients being, the lower the incidence will be. These features may make tumor patients less susceptible to symptomatic DVT[26]. It may also explain why asymptomatic DVT is not completely parallel to symptomatic DVT.

Factors accounting for VTE in patients with musculoskeletal tumors have not yet clearly understood. Several studies tried to investigate potential factors influencing VTE in patients with musculoskeletal tumors. However, no consensus was reached. Our study showed no statistically significant association between age (divided as <60y or >60y), BMI, tumor history, location and the development of VTE or DVT. (Table 2)

One study from Linn et al showed that there was no obvious risk factor associated with the incidence of DVT following cancer resection. Factors investigated in their study included age, gender, surgical procedures, tumor pathology, tumor location and pathological fracture[9]. Another study from Nathan et al including 87 patients with a malignant tumor showed anatomic location had a link with a significant risk of DVT [14]. In addition, Anderson et al [27]and Sakon et al[28] have proved a relationship between increasing age and a higher risk of VTE, which agrees with the results from Yamaguchi et al[29] who also found that only age ≥70 years increased the risk of DVT. On the contrary, several studies did not regard age as one important risk factor for the development of VTE [9–11, 14]. It has been reported that the application of peri-operative chemotherapy in tumor patients increased the risk of VTE. Heit et al [24]reported that cancer patients receiving chemotherapy had a 6.5 times risk of VTE compared with those free of cancer. Mitchell et al[11] reported a 4% rate of clinically evident deep venous thrombosim and a 1.2% overall rate of pulmonary embolism in a cohort with 252 patients undergoing Lower-Extremity Endoprosthetic Arthroplasty. One important finding from this study was that the majority of the events occurred prior to definitive surgery which might lead one to question the optimal anticoagulant therapy strategy for such susceptible populations. Barbara et al [30] observed that patients who have malignant diseases experienced a three- to six-times higher risk for both VTE and major bleeding compared with patients without malignancy. For metastatic bone tumors, there have been some studies showing a high risk for venous thrombosis or embolism [31]. For the routine use of chemoprophylaxis, Ramo et al [13] have performed a retrospective comparative review of 423 patients who had undergone prosthetic reconstruction after resection of lower-extremity musculoskeletal tumors. They didn’t find any significant difference between the use of any or no chemoprophylactic agent and the incidence of VTE events.

Actually, it is difficult to compare the incidence of VTE from different studies, as the basic information of the patients, the features of tumor, the method of DVT screen and the management of VTE prophylaxis differed between studies. This study specifically focused on the risk factors of VTE in patients with musculoskeletal tumors of knee for the first time.

## Strengths and Limitation

Strengths of the study include the large sample size in our series (both musculoskeletal tumors patients and Osteoarthritis patients). In addition, we used standardized screening methods, thus minimizing potential bias which may confound the results. In our study, the imaging studies were only reviewed by senior image doctors to ensure the quality of the ultrasound investigations. Considering the complex results of VTE, we divided it into asymptomatic, symptomatic DVT and PE. Such distinction may attribute to clinical practice as symptomatic DVT and PE would have more clinical value. For the suspects, except ultrasound examination, venography was also applied to ensure the result. By all the means, we hope to make our conclusion more convictive.

Limitations include only collecting limited information about patients’ characteristics and treatment. We failed to track some more important details like surgical time, strategy of anesthesia, blood loss and different patterns of pharmacologic thromboprophylaxis. All the above mentioned factors may have a potential influence for the development of VTE. In addition, we only used standard Doppler ultrasonography as the screen method for asymptomatic DVT and therefore we may have missed some potential DVTs because we did not routinely perform venography which had been recognized as the gold standard[32]. Likewise, we can provide data only on symptomatic PE because we did not perform computed tomography/angiography routinely on our patients unless symptoms were present.

## Conclusion

The non-significant incidence of clinically systemic DVT and PE between the tumor group and OA group suggests that using the same form of chemical or mechanical thromboprophylaxis after artificial joint replacement surgery will not show any difference between two groups. For patients with musculoskeletal tumors, some previous recognized potential risks showed no relationship with the development of VTE. Higher level clinical trials are needed to illustrate the relationship between the risk factors and VTE.

## Supporting Information

S1 Data Demographics and primary outcome of patients with musculoskeletal tumors around the knee. (XLSX)
